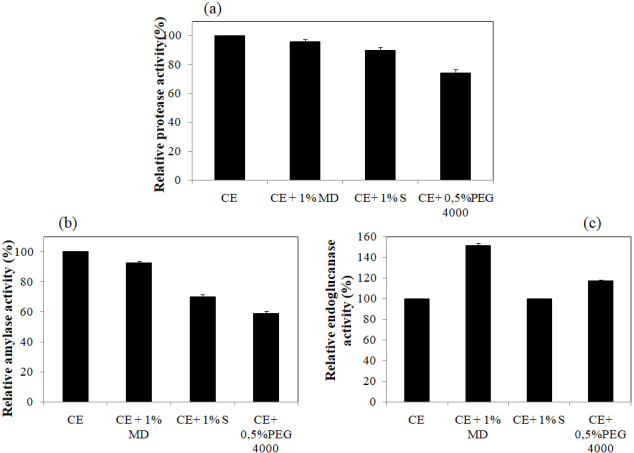# Stable and effective eco-enzyme cocktails in powder and liquid form of *Stachybotrys microspora* used as detergent additives

**DOI:** 10.1016/j.heliyon.2024.e25610

**Published:** 2024-02-07

**Authors:** Ines Ben Hmad, Ali Gargouri

**Affiliations:** Laboratory of Molecular Biotechnology of Eukaryotes, Centre of Biotechnology of Sfax (CBS) University of Sfax, B.P “1177” 3018, Sfax, Tunisia

**Keywords:** Green detergent, Enzymes stability, Biodegradation, Lignocellulosic waste, Cleaning efficiency

## Abstract

**Objective:**

The present work aims to optimize fermentation parameters for the simultaneous production of eco-enzymes: proteases, amylases, and endoglucanases from the same fungus *Stachybotrys microspora*, and to evaluate their stability in free form and formulated in lye as detergent additives.

**Methods:**

Initially, enzyme cocktail production was assayed in a medium comprising inexpensive waste biomass. Using the best substrate, we investigated the effect of its different concentrations and the NaCl concentration on the three enzymes co-production. Next, we studied the effect of several additives on the storage stability of the lyophilized enzyme cocktail (powder in liquid forms) free and incorporated in commercial laundry detergent. Finally, the washing efficiency analysis of the newly formulated enzyme cocktail was evaluated on dirty tissue pieces with different stains.

**Results:**

The highest enzymatic cocktail production was achieved at 30 °C for 96 h after adding 0.1% NaCl and 1.5% wheat bran as waste biomass in the basal culture medium. The effect of adding maltodextrin, sucrose, or polyethylene glycol 4000 during freeze-drying showed that maltodextrin is the best additive to protect the activities of proteases, amylases, and cellulases of liquid and powder enzyme form. Additionally, the liquid formulation of these enzymes showed excellent stability and compatibility with 1% maltodextrin and 10% glycerol. Interestingly, we have developed a new formulation of an enzyme cocktail (liquid and powder) stable and highly compatible with detergents. Comparing the washing performance of different formulations containing our enzyme cocktail to commercial ones showed significantly better removal of different types of stains.

**Conclusions:**

This research shows a cost-effective approach to simultaneously produce proteases, amylases, and endoglucanases from *Stachybotrys microspora* that could be considered a compatible detergent additive in the green detergent industry.

## 1 Introduction

Green detergent is based on eco-friendly enzymes in its composition; these enzymes are characterized by their biodegradability, environmental friendliness, low toxicity, and non-corrosiveness [1,2]. Additionally, these compounds are active at lower wash temperatures and shorter agitation times with improved detergent performance [3,4].

The detergent industry is considered the most enzymes-consuming sector. The international detergent enzymes market reached 1.8 million dollars in 2018 with a compound annual growth rate of 11.3% since 2013 [5]. Various microbes such as filamentous fungi, yeasts, and bacteria are the source of a large number of enzymes that have been recognized as a result of extensive research. Nowadays, they are used in many industrial applications [6,7].

Generally, proteases (EC 3.4.21–24 and 99), lipases (EC3.1.1.3), amylases (EC 3.2.1.1), and endoglucanases (EC 3.2.1.4) are used as additives in detergent formulations to remove dirt based on proteins, fatty residues, polysaccharides and respectively improve the cleaning, the brilliance of the colors and the softening of the fabrics [[8], [9], [10], [11]].

Since proteins have very sensitive structures, they must be stabilized and protected by a support material [12]. Several studies have shown that the stability of encapsulants such as corn syrup, starch, maltodextrin, inulin, polyethylene glycol (PEG), and arabic gum, can enhance the stability of compounds. Sensitive chemicals in coating materials are encapsulated in these capsules [13,14].

Moreover, the effectiveness of the enzyme depends on its compatibility with other compounds in the detergent formulation such as anionic surfactants, bleaches, and builders [15]. Indeed, the interaction between surfactants and enzymes can modify the structures of proteins due to various hydrophilic and hydrophobic groups and different charges [3,11]. So, a major problem in formulating enzymatic detergents is that of ensuring enzyme stability during storage. Furthermore, the degreasing efficiency is affected by the concentration of detergent in the washing solution, the type and concentration of surfactants, as well as enzymes that are part of the product [16].

Currently, detergent applications require the combined use of microbial enzyme cocktails. However, the key problem for the use of an enzyme cocktail comprising proteases, amylases, and endoglucanases is the lysis of the two latter enzymes by the proteases. Several detergent formulations are based on the addition of proteases, amylases, and endoglucanases, produced from various microbial strains, which increases the risk of attack of enzymes by proteases. To solve this problem, research has proposed the addition of a protease stabilization system. For example, the system containing sodium borate forms an enzyme-inhibitor complex which will be dissociated after dilution during washing [17]. For environmental reasons, the use of borate is reduced. Another study has shown that the addition of a reversible protease inhibitor (peptide) in the formulation of liquid laundry detergent has an affinity for proteases. Indeed, the formation of a protease-inhibitor complex will prevent the protease from degrading any other enzyme. Once diluted, the protease will be reactivated [18,19].

Thus, there is generally a demand for new microbes capable of simultaneously producing enzymes for detergent applications, in particular proteases, amylases, and endoglucanases, and in this case, proteases would not attack the other enzymes because they are derived from the same source [20].

Moreover, the overall production cost of enzymes is another major issue against its use in industries. Indeed, the cost of the growth medium is estimated to be about 40% of the overall manufacturing price of commercial enzymes [21]. Therefore, the formulation of detergent-suitable enzymes in a single fermentation with cheap carbon sources such as agro-industrial products is a growing need to reduce the costs of a production medium [22].

*Stachybotrys microspora* is a local fungal strain that produces alkaline cellulases and grows over a wide pH range [23] while most fungi have an optimal acidic pH within 4–6 [24].

More interestingly, this fungus secretes two halophilic endoglucanases (EG1 and EG2) that are active in the presence of NaCl and the presence of tap water [25,26]. Moreover, the effect of NaCl on purified endoglucanase from *Stachybotrys microspora* (EG1) revealed that the activity was enhanced by increasing salt concentration, with the optimum of activity being reached at 5 M of NaCl (152%), which is the highest optimum ever described for fungal endoglucanases. Moreover, the activity optima shifted from pH 7 and 50 °C to pH 8 and 70 °C in the presence of 5 M NaCl. Additionally, this enzyme exhibited a high resistance to SDS, a very potent detergent and strong denaturant of enzymes [25]. Furthermore, EG1, a halo-alkali tolerant endoglucanase, is compatible with the detergent formulation. These enzymes retained their activity in the presence of some commercial detergents such as OMO (91.4%), Sotup (liquid detergent) (94.6%), and New Det (85.6%). Such high resistance to commercial detergents makes this alkalophilic cellulase useful in the detergent and laundry industry [25]. These properties are exceptional in fungal cellulases. In addition to cellulolytic enzymes and in particular several β-glucosidases [22,27], *S. microspora* secretes other hydrolytic activities such as proteases and chitinases [28].

The present work aims to determine the fermentation parameters for the simultaneous production of three enzymes: proteases, amylases, and endoglucanases from *Stachybotrys microspora* and to study the stability and compatibility of the enzymatic cocktail formulated in liquid and powder form free and in the existence of commercial detergent bases. It also describes the effectiveness of our formulated enzyme cocktail in removing various types of stains.

## 2 Materials and methods

### 2.1 Materials

This work was carried out on the mutant A19 from the *Stachybotrys microspora* (N1) strain [29]. N1 is a filamentous fungus. It was isolated by our laboratory group and was identified as *Stachybotrys microspora* by the Centraalbureau voor Schimmelcultures, the Netherlands [29].

The additives maltodextrin (MD) (DE 13–17), sucrose (S), glycerol, and sorbitol were purchased from Sigma-Aldrich (St- Louis, USA). Polyethylene glycol 4000 (PEG) was purchased from Bio Basic (Markham, Canada). Casein from bovine milk, starch from potato, and carboxymethyl-cellulose were purchased from Sigma–Aldrich (St-Louis, USA). Wheat bran and gruel were purchased from a Tunisian food production company. Olive pomace was purchased from an olive press in Sfax-Tunisia. Soybean meal and chicken feathers were purchased from local farmers. As well as wood sawdust was purchased from a local carpenter, Sfax-Tunisia.

### 2.2 Methods

#### 2.2.1 Growth medium conditions

*Stachybotrys microspora* was cultured at a temperature of 30 °C, pH 7, and with shaking at 150 rpm. The initial growth medium for enzymatic cocktail production from this fungus was composed of a modified Mandels medium (g/L) [30]: 2.0 KH_2_PO_4_, 1.4 (NH_4_)_2_SO_4_, 1.0 yeast extract, 0.3 CaCl_2_·2H_2_O, 0.3 MgSO_4_·7H_2_O, 1.0 mL Tween 80 and a 1 mL trace element solution comprising 1.6 MnSO_4_, 2 ZnSO_4_, 0.5 CuSO_4_, and 0.5 CoSO_4_, supplemented with 10 g/L carbon source (wheat bran, gruel, olive pomace, soybean meal, chicken feathers and wood sawdust).

Each of the inexpensive carbon sources was applied individually at 10 g/L, to study the effects of various substrates on the production of proteases, amylases, and endoglucanases. The study of the concentration of the most readily available carbon source, wheat bran (WB), on the co-production of the enzymatic cocktail was evaluated by including 10, 15, and 20 g/L of WB in the growth medium under the above conditions.

Enzymatic cocktail was obtained by centrifuging at 4500 rpm*,* for 15 min at 4 °C to remove mycelium. The supernatant specimens were analyzed for proteases, amylases, and endoglucanases activities.

#### 2.2.2 Protease activity assay

Protease activity was determined according to the method of Kembhavi with casein as substrate [31]. Properly diluted crude enzyme (0.5 mL) was added in 0.5 mL of 0.1 M glycine-NaOH at pH 9 including 10 g/L casein. The mixture was incubated for 15 min at 50 °C. 0.5 mL of 20% trichloroacetic acid (TCA) was added to stop the reaction. The reaction medium was maintained at room temperature for 15 min. The supernatant was obtained by centrifugation at 13,000 rpm, for 15 min at 4 °C Measurement of absorbance was determined at 280 nm. The blank measurement was set using heat-denatured enzymatic preparation and substrate to eliminate any potential interference during spectrophotometer measurements. One unit of protease activity was determined as the amount of enzyme needed to release 1 μg of tyrosine per mL in 1 min under the experimental tests (1).

(1) $$\text{Protease}\;\text{activity}\;\left(U/\text{mL}\right)=\frac{\text{Absorbance}\times D\times d}{t\times 0,0022}$$

**d**: Dilution of the enzyme in the final volume of the reaction medium.

**D**: Dilution of the enzyme.

**t**: Reaction time in minutes.

**0.0022:** 1 μg/mL of tyrosine corresponds to an absorbance at 280 nm of 0.0022.

##### 2.2.2.1 Protease activity in detergent assay

The protease activity present in the detergent was assayed by the method described in Lin [31] with N, Ndimethylated casein (DMC) being used as a substrate. The crude enzyme preparation (0.5 mL), was added in 1 mL detergent base (detergent without enzymes added) and mixed with 2 mL 50 mM Borate-NaOH buffer (pH 9) including 0.4% DMC and 0.25 mL of 5% 2,4,6-trinitrobenzene sulfonic acid as a color indicator [32]. The reaction medium was incubated at 50 °C for 25 min. After that, it was stopped by the addition of 2.5 mL of water for 15 min. The supernatant was obtained by centrifugation at 13,000 rpm*,* for 15 min at 4 °C to remove the precipitate. Absorbance was determined at 450 nm. One unit of protease activity was calculated as the amount of enzyme needed to release 1 μmole of peptide bond from N, Ndimethylated casein per minute under the experimental conditions used.

#### 2.2.3 Amylase activity assay

Amylase activity was evaluated by using potato starch as a substrate. The crude enzyme (0.5 mL) was mixed with the solubilized substrate of 1% (w/v) to 0.1 M acetate buffer pH 5. The incubation of the reaction medium lasted 30 min at 50 °C. The amount of reducing sugars released was determined using the 3,5-dinitrosalicylic acid (DNS) way [33]. The reaction mixture including 3 mL of DNS was heated for 10 min. Then, 20 mL of redistilled water was added to the mixture. Absorbance was determined at 550 nm, to measure the enzyme activity. Blanks were prepared for every sample to remove the non-enzymatic liberation of reducing sugars. They were set using heat-denatured enzymatic preparation and substrate to eliminate any potential interference during spectrophotometer measurements.

One unit (U) of amylase activity was determined as the quantity of enzyme releasing 1 μmol of glucose per minute under the assay conditions (2).

(2) $$Amylase\;activity\;\left(U/mL\right)=\frac{Absorbance\;at\;550\;nm\;x\;DF\;x\;\left(VR/VE\right)\;*10-3}{Incubation\;time*180\;x\;10-6}$$

**DF:** DNS factor: the DNS factor is determined from the calibration curve produced with D-glucose.

**V**_**R**_**:** the reaction volume.

**V**_**E**_**:** The volume of the enzyme test portion.

**180 g/mol:** Molecular mass of glucose.

**Incubation time:** 30 min.

#### 2.2.4 Endoglucanase activity assay

Endoglucanase activity was assayed using carboxymethyl-cellulose (CMC) as a substrate. The reaction mixture including 0.5 mL of 1% CMC and 0.5 mL of crude enzyme diluted with 50 mM citrate buffer at pH 4.8 was incubated at 50 °C for 30 min. After adding 3 mL of DNS, the reaction mixture was heated for 10 min. Finally, the solution was diluted by adding 20 mL of distilled water, and the absorbance was measured at 550 nm. The “blanks” were set using heat-denatured enzymatic preparation and substrate to eliminate any potential interference during spectrophotometer measurements [33]. The unit of enzymatic activity was defined as the quantity of enzyme demanded to release 1 μmol of reducing sugar per minute under test conditions (3).

(3) $$Endoglucanase\;activity\;\left(U/mL\right)\left(\frac{}{}\right)=\frac{Absorbance\;at\;550\;nm\;x\;DF\;x\;\left(VR/VE\right)*10-3}{Incubation\;time*180\;x\;10-6}$$

**DF:** DNS factor: the DNS factor is determined from the calibration curve produced with D-glucose.

**V**_**R**_**:** the reaction volume.

**V**_**E**_**:** The volume of the enzyme test portion.

**180 g/mol:** Molecular mass of glucose.

**Incubation time:** 30 min.

#### 2.2.5 Lyophilization process

The lyophilization or freeze-drying of the crude enzyme preparation was carried out using a vertical Biobase freeze-dryer machine (temperature = – 40 °C). Previous to lyophilization, 1% sucrose (S), 1% maltodextrin (MD), or 0.5% polyethylene glycol (PEG) 4000 was mixed with the crude enzyme. As a control, a crude enzyme solution was freeze-dried without additives. The lyophilized enzyme cocktail powder was named CEP. The liquid form (CEL) consists of the suspension of CEP in a phosphate buffer 20 mM, pH7 which has been stored at room temperature for two months.

#### 2.2.6 Storage stability

The stability of the lyophilized enzymatic cocktail (liquid and powder) with the best additives was investigated during storage at room temperature in solid laundry bases (SLB) and liquid laundry bases (LLB) from Sodet (Tunisia and detergent company of Tunisia Sfax-Tunisia). Note that “bases” mean “without enzymes added”. The lyophilized proteases, amylases, and endoglucanases (2000 U, 50 U, and 6 U, respectively) in solid form or liquid form were added to solid laundry bases or liquid laundry bases and maintained at room temperature. Samples were withdrawn at a fixed period to examine residual activity (proteases, amylases, and endoglucanases). The detergents were mixed with tap water to obtain a concentration of 7 mg/mL.

#### 2.2.7 Effect of glycerol and sorbitol on the stability of liquid enzyme cocktail

The effect of glycerol and sorbitol on liquid enzyme cocktail stability was investigated by mixing the lyophilized enzymatic cocktail containing 1% maltodextrin in liquid form with 10% glycerol or 1% sorbitol purchased from Sigma-Aldrich (St- Louis, USA) and maintained at ambient temperature. Samples were taken at fixed periods to measure the residual activities. The solutions of lyophilized enzymatic cocktail liquid form (CEL) and lyophilized enzymatic cocktail containing 1% maltodextrin (CEL + MD), in 20 mM phosphate buffer without a polyol were used as the control.

#### 2.2.8 Cleaning performance assays

Dirty tissue pieces (5 cm × 5 cm) with different stains such as tomato sauce, human blood, and chocolate yogurt (the same quantities of dirt were deposited on the cotton fabrics of the same size and then they were dried) are used to simulate the cleaning conditions. This was done to determine the efficiency of the lyophilized enzyme cocktails with 1% maltodextrin in powder form (CEP + MD) and liquid form with 1% maltodextrin and mixed with 10% glycerol (CEL + MD + 10% glycerol) as detergent bio compounds compared to the Totalase T (commercial enzyme cocktail powder including proteolytic, amylolytic and cellulolytic enzymes) and Medley brilliant 100L (commercial enzyme cocktail liquid including proteolytic, amylolytic and cellulolytic enzymes) from Novozymes.

After drying, the dirty tissue pieces were diluted in tap water, mixed with the same volume of enzyme cocktails in powder form (CEP) and liquid (CEL + MD+ 10% glycerol) or commercial enzymatic cocktails, and incubated (200 rpm) in different wash processes at 40 °C for 30 min in beakers (1L) including solid laundry base (SLB) and liquid laundry base (LLB) (7 mg/mL). Control tissues, treated only with detergent bases were conducted under the same condition.

#### 2.2.9 Statistical analysis

Each assay was performed in duplicate. The standard deviations for each experimental result were calculated using the Microsoft Excel Software.

## 3 Results and discussion

### 3.1 Optimization of proteases, amylases, and endoglucanases co-production by Stachybotrys microspora

The microbial strain has specific parameters for optimal co-production of enzymes. In this respect, different sources of carbon and NaCl have been tested to economically optimize a growth medium allowing the maximum co-production of proteases, amylases, and endoglucanases by the fungal strain of *Stachybotrys microspora*. Initially, enzyme production was assayed in a medium comprising six different inexpensive and readily available substrates (wheat bran, gruel, olive pomace, soybean meal, chicken feathers, and wood sawdust) at 10 g/L. As shown in Table 1, the optimal carbon source for the co-production of proteases and endoglucanases was wheat bran (356.82 U/mL and 0.52 U/mL, respectively) after 72 h and 120 h of cultivation, respectively. The amylase activity was maximum on gruel (5.75 U/mL), then by wheat bran (4.03 U/mL) after 120 h of cultivation. The highest levels of co-produced proteases, amylases, and endoglucanases were reached between 72 h and 120 h, beyond which a progressive decrease in the level of enzymes was observed which could be due to the release of inhibitory metabolites during the stationary phase. In addition, the accumulation of certain products of enzymatic reactions leads to a repression effect [34]. Many studies use a variety of organic sources as a substrate in enzyme production such as wheat bran, oat flour production, and potato peel powder [[34], [35], [36], [37]]. Indeed, the production of enzymes is inducible and strongly affected by the types of carbon sources (induced or repressed). Additionally, wheat bran is characterized by high water-holding capacity and high porosity allowing easy penetration of fungal hyphae and it has been reported to induce a variety of hydrolases [38]. Many articles found similar conditions and showed that the highest production of amylases and endoglucanases by the fungal strain was observed after 120 h of incubation [34,39]. The similar maximum production of fungal proteases was at 72 h of incubation [40,41].

**Table 1:** Effects of various cheap carbon sources (waste biomass (10 g/L)) on co-production of proteases, amylases and endoglucanases by *Stachybotrys microspora,* after 72 h and 120 h of cultivation.

Cheap carbon sources (10 g/L)	Residual enzyme activity (U/mL)		
	Proteases	Amylases	Endoglucanases
**Gruel**	325.64 ± 2.312	5.75 ± 0.070	0.34 ± 0.007
**Olive pomace**	172.50 ± 4.313	0.54 ± 0.014	0.44 ± 0.014
**Soybean meal**	259.86 ± 3.153	2.11 ± 0.021	0.37 ± 0.014
**Chicken feathers**	294.45 ± 5.020	1.08 ± 0.113	0.08 ± 0.005
Wheat bran	**356.82 ± 7.637**	**4.03 ± 0.028**	**0.52 ± 0.012**
**Wood sawdust**	151.82 ± 2.566	0.26 ± 0.009	0.49 ± 0.014

**Fig. 1:**
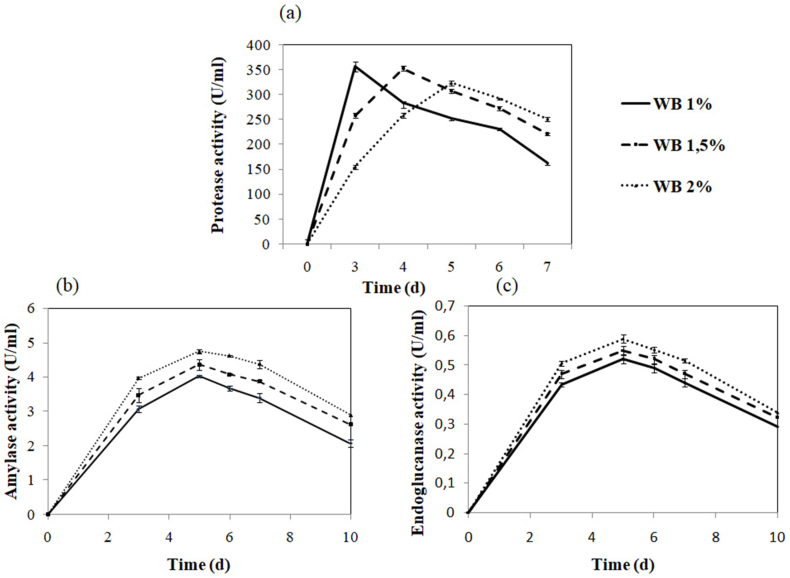
Effects of different concentrations of wheat bran (WB) on (a) proteases, (b) amylases, and (c) endoglucanases co-production by *Stachybotrys microspora*.

Since the best substrate (wheat bran), we investigated the effect of its different concentrations on the three enzymes' co-production. Fig. 1 shows that the best wheat bran (WB) concentration to co-produce proteases, amylases, and endoglucanases was 15 g/L after 96 h cultivation (353.18 U/mL, 4.20 U/mL, and 0.53 U/mL), respectively (Fig. 1a, b, c). Although different enzyme components were maximally secreted at different incubation times, 96 h of incubation was selected for the following studies. At this incubation time, all three enzymes showed considerable yields with the least compromise.

Moreover, the addition of NaCl at 0.1% (v/v) in the medium containing 15 g/L of wheat bran significantly improved proteases, amylases, and endoglucanases co-production by 1.22 folds, 1.23 folds, and 1.6 folds, respectively (Table 2). Previous studies [[42], [43], [44]] have reported that NaCl enhances enzyme secretion. Indeed, this salt exhibits a crucial role in protecting and stabilizing proteins from inactivation [42,43]. This optimal condition of the culture medium will be applied to all subsequent works. Therefore, the *Stachybotrys microspora* can simultaneously produce an enzymatic cocktail without the degradation of enzymes by proteases.

**Table 2:** Effect of [NaCl] concentration in the growth medium containing 15 g/L of wheat bran on the proteases, amylases and endoglucanases co-production by *Stachybotrys microspora* after 96 h cultivation.

[NaCl] Concentration in the medium (%)	Relative activity (%)		
	Proteases	Amylases	Endoglucanases
0	100.0	100.0	100.0
0.05	109.0 ± 1.41	104.5 ± 0.70	127.5 ± 3.53
**0.1**	**122.0 ± 2.82**	**123.5 ± 2.12**	**158.5 ± 2.12**
0.5	87.5 ± 3.53	121.0 ± 1.41	141.0 ± 1.41
1	37.5 ± 3.53	96.5 ± 2.12	116.5 ± 2.12

### 3.2 Storage stability of the freeze-drying enzyme cocktail

Generally, application of enzymes in laundry detergents is limited because of their inactivation during storage. This work investigated the effect of several excipients on the proteolytic, amylolytic, and cellulolytic activity and storage stability of the lyophilized enzyme cocktail. The addition of maltodextrin (1%), sucrose (1%), or polyethylene glycol (0.5%) retained approximately 96% ± 1.41, 90% ± 2.12, and 74% ± 2.82 of the proteolytic activity, respectively (Supplementary data). The amylolytic activity preserved approximately 96% ± 1.41, 70% ± 1.41 and 59% ± 1.41, respectively. However, the incorporation of these additives improves the endo cellulolytic activity to 151.5% ± 2.12, 100%, and 117% ± 1.41, respectively (Supplementary data).

**Fig. 2:**
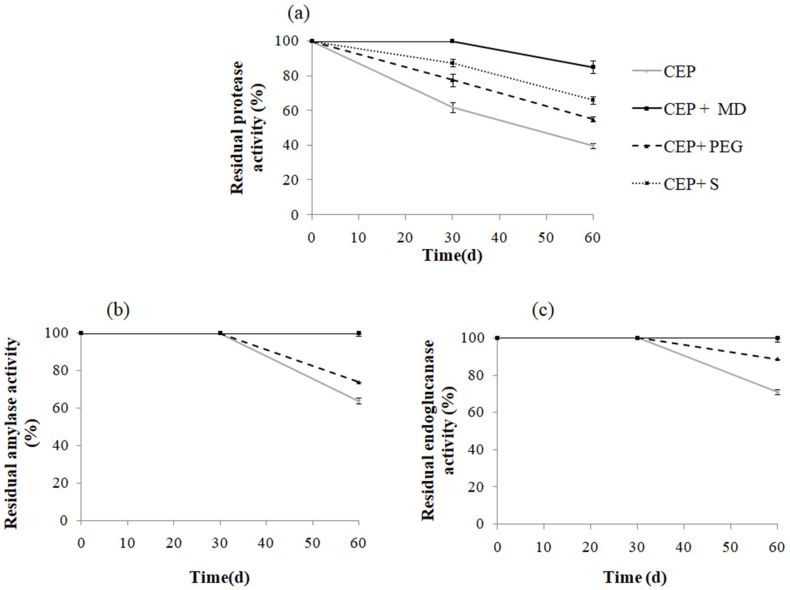
Stability of the enzyme cocktail powder form (CEP) free and with additives (MD: maltodextrin; PEG: polyethylene glycol 4000 and S: sorbitol) during storage at room temperature. Before incubation, the activity of each enzyme was taken as 100%. Values are the means of two independent experiments.

**Fig. 3:**
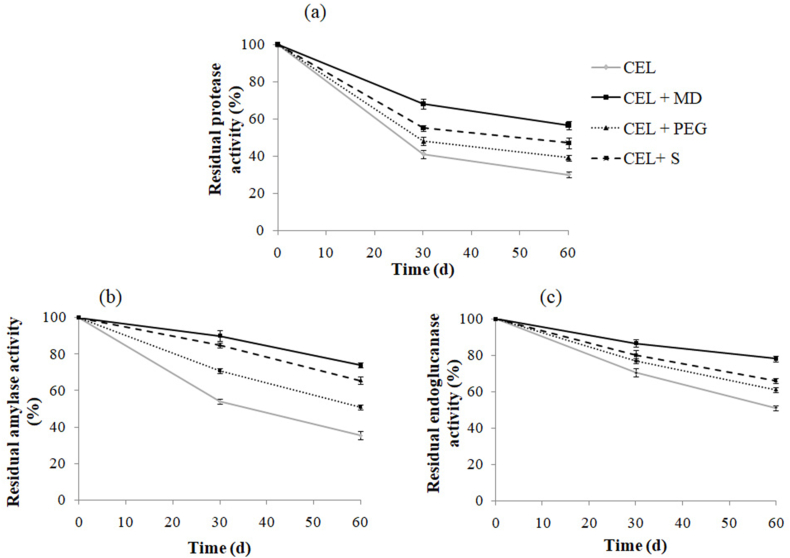
Stability of the enzyme cocktail liquid form (CEL) free and with additives (MD: maltodextrin; PEG: polyethylene glycol 4000 and S: sorbitol) when stored at room temperature. Before incubation, the activity of each enzyme was taken as 100%. Values are the means of two independent experiments.

Fig. 2, Fig. 3 showed previous excipients applied for lyophilization enhanced the stability of both powder form (CEP) (Fig. 2) and liquid (CEL) enzymes (Fig. 3). Between the excipients evaluated, maltodextrin gave the best effects. Similarly, Naganthran et al. showed the important role of maltodextrin in the stability of protease, amylase, and lipase [44].

In addition, the stability of the lyophilized enzymes with 1% maltodextrin (CEP + MD), exhibited extreme stability after incubation at room temperature for 60 days against 60, 36 and 30% for the control without additives (Fig. 2 a,b,c). Indeed, the use of maltodextrin in the formulation can decrease the water level of the dried powder in the freeze-drying process. Additionally, this drying agent can decrease the amount of free water and increase the amount of powder during the freeze-drying process. Thus, the powder obtained contains less water than the dried powder without maltodextrin [45]. The stability of proteins could be improved by covalently bonding polysaccharides by averting them from deployment [46].

However, CEL lost about 43, 26, and 22% of its original proteolytic, amylolytic, and endocellulolytic activity, respectively against 70, 64.5, and 50% for the control without additives (Fig. 3 a,b,c). Thus, the enzymatic cocktail powder exhibited excellent stability in comparison with the liquid form. The advantage of freeze-drying is that simultaneous freezing and drying generates a dry, active, and stable enzyme. Additionally, several studies have shown that the inclusion of additives such as polyols (an organic compound containing multiple alcoholic hydroxyl groups) and polysaccharides improves the stability of the protein during freeze-drying and storage in solid detergents [13,44,45].

Moreover, the performance of carbohydrates to protect the enzymatic activity of the dried product can be explained by the formation of a glassy matrix or by the replacement of water in the interactions with the protein [47]. Simončič and Lukšič suggest that hydrogen bonds between water molecules and proteins are replaced by hydrogen bonds between sugar hydroxyl groups and proteins [48].

### 3.3 Effect of polyols on the enzymatic cocktail liquid form stability

**Fig. 4:**
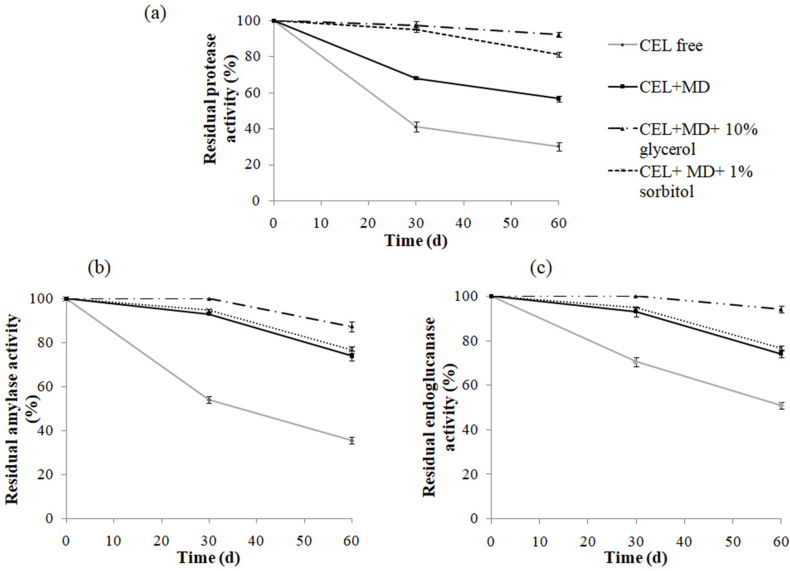
Effect of polyols (glycerol and sorbitol) on the enzyme cocktail liquid form (CEL) stability-free or with maltodextrin (MD). Values are the means of two independent experiments.

To improve the enzymatic cocktail lyophilized with maltodextrin liquid form stability, the effect of 10% glycerol or 1% sorbitol was investigated. Fig. 4 shows that glycerol is the best additive that increases the stability of the three enzymes in liquid form. Indeed, the enzymatic cocktail liquid form (CEL) containing both maltodextrin and glycerol lost only about 20, 8 and 10% of its original proteolytic, amylolytic, and endocellulolytic activity, respectively, after incubation at room temperature for 60 days (Fig. 4 a,b,c). Several studies have shown that glycerol improves the stability of proteins in aqueous solution [48,49]. These interactions shift the native proteins into more compact conformations. Glycerol prevents protein aggregation by acting as an amphiphilic interface between the hydrophobic surface and the polar solvent which inhibits protein unfolding [49].

The sorbitol improves only protease activity (Fig. 4a). In the presence of maltodextrin and sorbitol (MD + sorbitol), amylolytic and endocellulolytic activity showed similar behavior to maltodextrin alone (Fig. 4b and c). Therefore, the protein disaggregation can be explained by the favorite hydration of the enzyme after mixing with osmolyte, which produces intra-chain bonds that can favor equilibrium in the direction of active conformations [50].

### 3.4 Stability of enzyme cocktail within commercial laundry detergents

For applicability to detergent formulations, the compatibility of both liquid and powder forms of enzymes in laundry detergents was investigated. It was evaluated by monitoring the enzymatic stability of (protease, amylase, and endoglucanase) after incorporation into laundry detergent during storage. So, samples were withdrawn at a fixed time to examine the residual activity of enzymes.

**Fig. 5:**
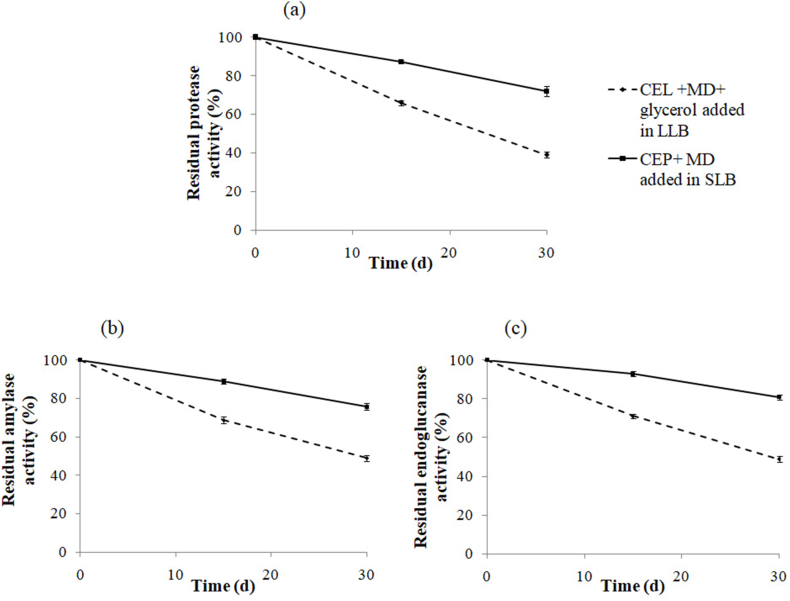
Study of both enzyme cocktail liquid form (CEL + MD + glycerol) and powder form (CEP + MD) stability during storage within liquid laundry base (LLB) and solid laundry base (SLB), respectively. The retained activity was evaluated at a fixed period. Before incubation, the activity of each enzyme was taken as 100%. Values are the means of two independent experiments.

Indeed, the stability of the enzyme cocktail lyophilized with 1% maltodextrin powder form (CEP + MD) is enhanced when stored in solid laundry base compared to the enzymatic cocktail liquid form with 1% maltodextrin and 10% glycerol (CEL + MD + Glycerol) when stored in liquid laundry base (Fig. 5a, b, c). Incubated for 30 days at room temperature, the enzymatic cocktail powder form retained 72%, 75% and 80% of the initial proteolytic, amylolytic and endocellulolytic activities, respectively (Fig. 5a, b, c). The protective effect can be explained by the enhancement of intramolecular hydrophobic bonding. In addition, water removal decreases protein movement, and inhibits conformational changes that cause their inactivation [51].

### 3.5 Washing efficiency analysis

**Fig. 6:**
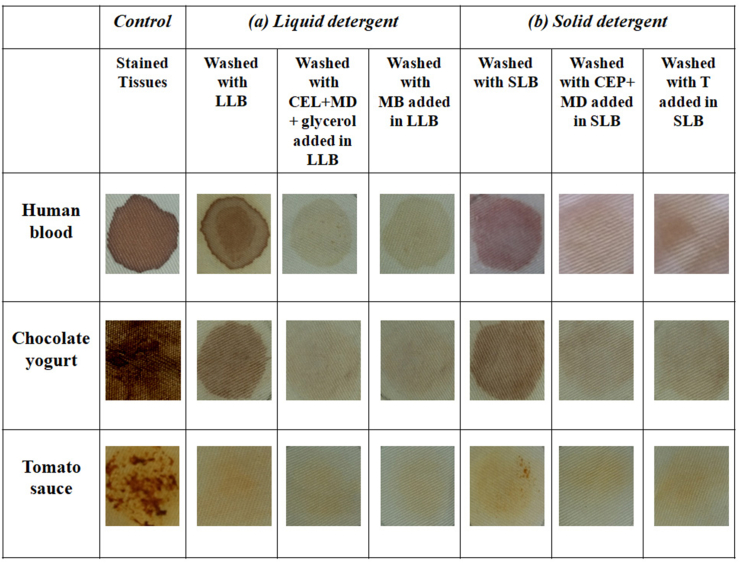
Wash performance analyses of (CEL + MD + glycerol) and (CEP + MD) from *Stachybotrys microspora,* Medley Brillant (MB) (commercial enzymatic cocktails liquid), and Totalase (T) (commercial enzymatic cocktails powder) on stained tissues in the presence of (a) liquid laundry base (LLB) and (b) solid laundry base (SLB).

Several soiled fabrics were analyzed under various conditions to evaluate the performance of the newly formulated enzyme cocktail in liquid (CEL + MD+10% glycerol) (Fig. 6a) and powder form (CEP + MD) (Fig. 6b) in removing stains from chocolate yogurt, human blood, and tomato sauce. We have determined the compatibility of enzyme cocktails as detergent bio compounds compared to the commercial enzyme (including proteolytic, amylolytic, and endo cellulolytic enzymes) and detergent without enzymes by washing efficiency analysis. Control tissues, treated only with detergent bases were conducted under the same condition. Fig. 6 shows that the “detergent compatible” green enzymes from *S. microspora* were distinguished by their potent ability to remove various stains from chocolate yogurt, human blood, and tomato sauce. Both powder and liquid formulations of the enzyme cocktail perfectly remove different types of stains after a washing treatment (30 min) in tap water including 7 mg/mL of solid laundry base or liquid laundry base. The results obtained with the newly formulated enzyme cocktail in both liquid (CEL + MD+ 10% glycerol) (Fig. 6a) and powder (CEP + MD) form (Fig. 6b) were better than those of commercial enzymes or detergents without enzymes (Fig. 6). These results show that the newly formulated *Stachybotrys microspora* enzyme cocktail in both liquid and powder form can improve the ability of detergent bases to remove various stains from washed tissues. Indeed, enzyme-based detergents can work efficiently, and accelerate the specificity and rate of a reaction by reducing the energy. The performance of the new enzymatic cocktail formulated from *Stachybotrys microspora* is manifested by the specific functions of three classes of enzymes: proteases hydrolyze peptide bonds and eliminate protein stains such as human blood, yogurt, and chocolate, amylases hydrolyze the 1,4-α-glycosidic bond and remove starch stains like sauces, pasta … and endoglucanases hydrolyze cellulose stains, remove broken cellulose fibers, clarify color and its redeposition. Several studies have reported the usefulness of microbial alkaline proteases in facilitating the removal of blood stains from cotton fabrics in the presence of detergents [52]. However, recent studies have reported the effectiveness of using enzyme cocktails in laundry detergents to remove stains rather than using enzymes individually. We can cite the enzymatic preparations of *Bacillus mojavensis* containing both α-amylase and protease activities [20], *Bacillus nealsonii* PN-11 containing protease and mannanase [53], and *Aspergillus niger* and *Aspergillus oryzae* containing protease, amylase, cellulase and lipase [54]. These studies show that the best results were obtained when the enzymes were used in combination. This is because detergent proteases or amylases work best by hydrolyzing insoluble proteins and starches present in stains. Initially, protein or starch soils are removed from the fabric surface by water alone or by detergent components. Depending on the size of the resulting fragments, they are either deposited into the tissue or solubilized in a bulk solution. Thus, the best detergent enzymes provide improved hydrolysis of the substrate allowing better stain removal and better anti-redeposition [55]. Furthermore, vegetable stains contained pigments which are associated with the fibrous materials either via physical binding (“sticking”) or via covalent bonds. Removing these plant stains can be very difficult because they are mainly attached to cellulose and hemicellulose fibers (non-starchy polysaccharides). Hence, for the most efficient removal of stains, endoglucanase and hemicellulase cut polymeric compounds into smaller pieces and therefore increase the solubilization of the fiber mass with its associated pigments [56]. Furthermore, detergents containing endoglucanases are making fiber modifications in the fabric to improve color brightness, softness, and particulate soil removal. Indeed, these enzymes can remove dirt particles from the inter-fibrillar spaces of the fabric [57].

The most recent innovation is to use combinations of enzymes in detergents. Indeed, several detergent formulations are based on the addition of proteases, amylases, and endoglucanases, produced from various microbial strains, which increases the risk of attack of enzymes by proteases. Whereas, the *Stachybotrys microspora* strain can simultaneously produce three classes of enzymes stable and effective. This is not the case for other microorganisms. In addition, this strain is halotolerant and can grow over a wide pH range up to pH 9. So, it can produce halo-alkalophilic enzymes, especially cellulases, while the majority of the commercial fungal endoglucanases are active at acidic pH. Furthermore, *Stachybotrys microspora* produces enzymes that are resistant to the presence of detergent. We have shown that commercial endoglucanase was much more inactivated in the presence of commercial laundry detergent than EG1 [25].

Moreover, the overall production cost of enzymes is another major issue against its use in industries. Indeed, the cost of the growth medium is estimated to be about 40% of the overall manufacturing price of commercial enzymes. Therefore, the formulation of detergent-suitable enzymes in a single fermentation with cheap carbon sources such as agro-industrial products is a growing need to reduce the costs of a production medium.

## 4 Conclusions

This work has shown that *Stachybotrys microspora* is an excellent co-producer of interesting proteases, amylases, and endoglucanases, in the presence of wheat bran as waste biomass and 0.1% NaCl after 96 h of cultivation. Furthermore, the addition of maltodextrin to the produced enzyme cocktail significantly improved enzyme stability, during lyophilization and storage. Moreover, the liquid form of the newly formulated enzymatic cocktail showed high stability with maltodextrin and glycerol. Interestingly, the powder form with maltodextrin showed the best stability and tolerability in a solid laundry base. Finally, the newly formulated enzyme cocktail in both forms liquid and powder has demonstrated good washing performance against several types of stains. Thus, the *Stachybotrys microspora* strain offers a cost-effective method to produce an enzyme cocktail defined as a “detergent compatible” compound, which is very practical in the detergent industry.

## Authors contributions statement

**Dr. Ines BEN HMAD**: Conceptualization and performed experiments, writing–original draft and writing–review and editing. **Ali GARGOURI**: Supervised responsibility for the research activity and writing–review, and editing. All authors read and approved the final manuscript.

## Funding statement

This work is executed under the MOBIDOC scheme (2018–2022), funded by the 10.13039/100009122Ministry of Higher Education and Scientific Research through the PromESsE project and managed by the National Agency for the Promotion of Scientific Research (ANPR).

## CRediT authorship contribution statement

**Ines Ben Hmad:** Writing – original draft, Conceptualization. **Ali Gargouri:** Visualization, Validation, Conceptualization.

## Declaration of competing interest

The authors declare that they have no known competing financial interests or personal relationships that could have appeared to influence the work reported in this paper.

## Figures and Tables

**figs1:**